# Supplementary material for: Getting More Out of Biomedical Documents with GATE's Full Lifecycle Open Source Text Analytics
Source: PLoS Comput Biol. 2013 Feb 7;9(2):e1002854. doi: 10.1371/journal.pcbi.1002854 (PMC3567135; doi:10.1371/journal.pcbi.1002854)
Supplement: Dataset S1 — GATE software. Dataset S1 bundled with this paper contains a distribution of GATE (or see http://gate.ac.uk/download/). (TGZ) [file pcbi.1002854.s001.tgz › plos-gate/doc/external-plugins.html]

- OrganismTagger
- Multi-lingual Noun Phrase Extractor (MuNPEx)
- Durm German lemmatizer
- XCES tools
- *Sen* wrapper (Japanese morphological analyser)
- Russian morph tagger
- String Annotation
- GATE Application Documentation
- VirtualCorpus - Directory- and JDBC Corpus LR
- BWP Gazetteer
- Apolda
- Reported Speech Tagger
- Keyphrase extraction module from SmILE

| OrganismTagger website download | | |
| --- | --- | --- |
| OrganismTagger | The OrganismTagger is a hybrid rule-based/machine-learning system that extracts organism mentions from the biomedical literature, normalizes them to their scientific name, and provides grounding to the NCBI Taxonomy database.  | |  |
| Multi-lingual Noun Phrase Extractor (MuNPEx) website download | | |
| Munpex | MuNPEx is a multi-lingual noun phrase (NP) extraction component developed for the GATE architecture, implemented in JAPE. It currently supports English, German, French, and Spanish (in beta). | en-np\_main.jape |
| Durm German lemmatizer website download | | |
| Durm German lemmatizer | The Durm German Lemmatization System consists of a number of GATE components and resources that perform morphological analysis and lemmatization for German nouns. |  |
| XCES tools website download | | |
| *Tools to handle documents conforming to the XML Corpus Encoding Standard (XCES) format, used by the American National Corpus. XCES is a way of encoding texts with standoff markup in XML.* | | |
| ANC Document | An XCES document. Allows loading of the document text, plus some or all of the sets of standoff markup associated with the document. | org.xces.gate.XCESDocument |
| ANC Load Standoff | Loads standoff annotations into an existing document. | org.xces.creole.LoadStandoff |
| ANC Save Content | Saves just the text content of a document to a file. This will work for any document - it is not specific to ANC/XCES documents. | org.xces.creole.SaveContent |
| ANC Save Standoff | Saves annotations from a Document to an XCES-compliant standoff markup file. | org.xces.creole.SaveStandoff |
| *Sen* wrapper (Japanese morphological analyser) website (in Japanese) | | |
| *Sen is a morphological analyser for Japanese. This is a wrapper to allow it to be used from GATE, you must also install sen itself. See the documentation (in Japanese) for details.* | | |
| Sen Wrapper | Morphological analyser for Japanese | jp.co.ditlab.jgate.SenWrapper |
| Russian morph tagger website download | | |
| *Provides GATE wrapper for mystem russian morphological parser. Allows to execute native analyzer, parse output, and assign morpho features as GATE annotations for the document.* | | |
| Russian MorphTagger | MorphTagger for russian language, based on MyStem Yandex' parser | ru.itbrains.gate.morph.MorphTagger |
| String Annotation Plugin  website download | | |
| *Processing resources for directly annotating the string content of a document.* | | |
| Extended List Gazetteer | Extended version of the GATE Default List Gazetteer. In addition to the features of the original, built-in version of the List Gazetteer, this version provides features for more powerful matching of partial words and annotating prefixes and suffixes as well as more versatile handling of word boundaries and whitespace. | at.ofai.gate.extendedgazetteer.ExtendedGazetteer |
| Simple Regexp Annotator | Use rules based on Java regular expressions to annotate the document content. | at.ofai.gate.regexpannotator.SimpleRegexpAnnotator |
| AppDoc — GATE Application Documentation  website download | | |
| *A plugin that generations documentation from your application/pipeline/gapp files in various formats. In addition it provides new visual resources in the GATE Developer GUI to add author, version and documentation comments to pipelines and processing resources.* | | |
| AppDoc | Visual resource for adding author/version/comment to pipelines and processing resources | at.ofai.gate.appdoc.AppDoc |
| AppDocGen | Visual resource for selecting a documentation template and generating documentation files | at.ofai.gate.appdoc.AppDocGen |
| VirtualCorpus — Directory- and JDBC Corpus LRs  website download | | |
| *A plugin that provides two new corpus language resources, DirectoryCorpus for directly using files in a directory through a corpus LR and JDBCCorpus for directly using documents stored in a field of a JDBC database table.* | | |
| DirectoryCorpus | Language resource for accessing GATE XML files in a directory directly via a corpus resource | at.ofai.gate.virtualcorpus.DirectoryCorpus |
| JDBCCorpus | Language resource for accessing GATE XML documents stored in a field of a JDBC database table directly via a corpus resource | at.ofai.gate.virtualcorpus.JDBCCorpus |
| BWP Gazetteer  website download | | |
| *This plugin provides an approximate gazetteer for GATE, based on Levenshtein's Edit Distance for strings. Its goal is to handle texts with noise and errors, in which GATE's default gazetteers may have difficulties.* | | |
| BWP Gazetteer | Extended version of the transducer-based List Gazetteer |  |
| Apolda  website download | | |
| *Annotates documents like a gazetteer, but takes the terms from OWL annotation properties in an ontology, rather than from a separate list of terms.* | | |
| Apolda Ontology Annotator | Ontology-based lookup taking terms from properties in the ontology. | telin.Apolda |
| Reported Speech Tagger  website download | | |
| *Automatically detects and tags reported speech constructs, in particular the source, reporting verb and content.* | | |
| Reporting Verb marker | JAPE transducer which tags reporting verbs |  |
| Reported Speech finder | JAPE transducer which tags reported speech |  |
| Keyphrase Extraction Module  website download | | |
| *Keyphrase extraction and language identification.* | | |
| FrequencyAnalyser |  |  |
| KeywordAnalyser |  |  |
| LanguageIdentification | identifies the language of a document using character n-grams |  |
| POSTagMapper |  |  |
| SimpleNounChunking |  |  |
| StopwordMarker |  |  |

---
